# Repertoire Analysis of B-Cells Located in Striated Ducts of Salivary Glands of Patients With Sjögren's Syndrome

**DOI:** 10.3389/fimmu.2020.01486

**Published:** 2020-07-14

**Authors:** Annie Visser, Gwenny M. Verstappen, Bert van der Vegt, Arjan Vissink, Richard J. Bende, Hendrika Bootsma, Nicolaas A. Bos, Frans G. M. Kroese

**Affiliations:** ^1^Department of Rheumatology and Clinical Immunology, University of Groningen and University Medical Center Groningen, Groningen, Netherlands; ^2^Department of Pathology and Medical Biology, University of Groningen and University Medical Center Groningen, Groningen, Netherlands; ^3^Department of Oral and Maxillofacial Surgery, University of Groningen and University Medical Center Groningen, Groningen, Netherlands; ^4^Department of Pathology, Academic Medical Center and University of Amsterdam, Amsterdam, Netherlands

**Keywords:** Sjögren's syndrome, B-cells, FcRL4, IGHV repertoire, rheumatoid factor, salivary gland, parotid gland, MALT lymphoma

## Abstract

A major complication of primary Sjögren's syndrome (pSS) is development of mucosa associated lymphoid tissue (MALT) B-cell lymphoma, particularly in salivary glands. These lymphomas express FcRL4 and are characteristically associated with lymphoepithelial lesions. Neoplastic B-cells may be derived from non-neoplastic glandular intraductal B-cells, also virtually all expressing FcRL4. A characteristic feature of MALT lymphomas is the production of rheumatoid factors (RFs), which are largely encoded by stereotypic immunoglobulin variable heavy chain (IGHV) sequences. The aim of this study was to examine whether there is a relationship between the intraductal and periductal B-cells and whether the intraductal B-cells are selected for RF. RNA was extracted from laser-microdissected infiltrated ductal areas and periductal infiltrates from frozen parotid gland tissue sections of 5 pSS patients. PCR amplified IGHV transcripts were cloned into pCR™4-TOPO vector and subsequently sequenced. Microdissected ducts yielded 96 unique IGHV sequences derived from intraductal B-cells, while 119 unique IGHV sequences were obtained from periductal infiltrates. No major difference in VH-gene usage was observed between intraductal and periductal B-cells. Nearly all (>90%) IGHV sequences derived from both intraductal and periductal B-cells were mutated. Clonal expansions as defined by shared VDJ rearrangements were also present among both intraductal and periductal B-cells: in total 32 clones were found, from which 12 were located within ducts, 15 in periductal areas, and five clones shared members in both areas. We observed 12 IGHV rearrangements encoding for RF sequences from which two were derived from intraductal B-cells and 10 from periductal B-cells. Nine RF sequences were part of a clone. Together these findings indicate that intraductal and periductal B-cells are closely related to each other. Intraductal B-cells are most likely derived from periductal B-cells. We did not obtain evidence that RF-specific B-cells are enriched within the striated ducts. We speculate that in principle any activated B-cell can enter the striated ducts from the periductal infiltrate, irrespective of its antigenic specificity. Within the ducts, these B-cells may receive additional activation and proliferation signals, to further expand at these sites and by acquisition of driver-mutations develop toward lymphoma.

## Introduction

Primary Sjögren's syndrome (pSS) is an autoimmune disease primarily affecting the salivary and lacrimal glands resulting in the characteristic complaints of dry mouth and dry eyes. B-cell hyperactivity is a hallmark of this disease as, amongst others, revealed by elevated levels of serum IgG and free light chains, presence of rheumatoid factor (RF) and anti-SSA/-SSB autoantibodies, and higher intrinsic levels of Bruton's tyrosine kinase (Btk), an enzyme critically involved in B-cell receptor signaling (1–3). The B-cell hyperactivity is also illustrated by the presence of clonal expansions of B-cells in labial and parotid glands of pSS patients (4–9). Chronic activation of B-cells probably also puts pSS patients at a high risk of neoplastic transformation. In fact, pSS patients have a 5–10% change of developing a non-Hodgkin B-cell lymphoma (NHL), in particular parotid gland mucosa-associated lymphoid tissue (MALT) B-cell lymphoma (3, 10).

Histologically, infiltrates of B- and T-lymphocytes as well as a wide variety of non-lymphoid cells, associated with the striated ducts, are usually seen in the salivary glands of pSS patients (11). Frequently the striated ducts comprise lymphoepithelial lesions (LELs). LELs are composed of proliferative metaplastic epithelial cells in combination with relative high numbers of intraepithelial lymphocytes. Although both B- and T-cells can be observed in the ductal epithelial layer, there is evidence that B-cells in the ducts, here referred to as intraductal B-cells, are involved in the formation of LELs (12). Importantly, B-cell depletion therapy with rituximab of pSS patients not only resulted in loss of B-cells from the ducts, but concomitantly also in amelioration of the LELs (13). The vast majority, if not all, B-cells within the LELs of both labial and parotid salivary glands of pSS patients express inhibitory Fc receptor-like protein 4 (FcRL4/IRTA1/CD307d) (14, 15). A relative small number of B-cells in the periductal infiltrate expresses FcRL4, and these cells are mostly located in close proximity to the epithelium. The relationship between the intraepithelial B-cells and the periductal B-cells is, however, not known. A relative large fraction (>20%) of the intraductal and a slightly smaller fraction of periductal FcRL4^+^ cells are actively dividing cells, as reflected by Ki-67 expression (15). Transcriptome analysis of FcRL4^+^ B-cells from parotid glands of pSS patients (16) revealed that these FcRL4^+^ B-cells show similarities in gene expression profile to chronically activated CD11c^+^T-bet^+^ memory B-cells, which were shown to be involved in the pathogenesis of systemic lupus erythematosus (17, 18).

The notion that parotid MALT B-cell lymphomas of pSS patients also express FcRL4, and that LELs are a characteristic feature of salivary gland MALT lymphomas, support the hypothesis that neoplastic MALT B-cells may arise from glandular FcRL4^+^ intraductal B-cells (15). The recent finding that several genes associated with lymphomagenesis are upregulated in FcRL4^+^ B-cells (16), strengthens this hypothesis. An important finding for MALT B-cell lymphomas is their frequent expression of antibodies with RF activity (19, 20). The transcripts of RF antibodies are frequently characterized by specific immunoglobulin heavy chain variable gene (IGHV) rearrangements. These rearrangements, result in amino acid sequences of the IGHV-complementarity determining region 3 (CDR3) region with reactivity for IgG-Fc, so-called stereotypic RF sequences (19, 21, 22). Although stereotypic RF sequences have been observed in labial gland and parotid gland biopsies of pSS patients, the proportion of these RF sequences is extremely low (21, 23–25). It might, however, be that the (non-neoplastic) FcRL4^+^ B-cells present in the striated ducts of non-lymphoma pSS patients are enriched in RF producing cells. In this study we therefore analyzed the IGHV repertoire of intraductal B-cells and B-cells located in the periductal areas and analyzed their VH-gene usage, mutation frequency, clonal expansion, and presence of stereotypic RF sequences.

## Materials and Methods

### Patients

Parotid gland biopsies of five patients fulfilling the ACR-EULAR criteria for pSS (26) were included in this study. Patient characteristics are summarized in Table 1. All patients were female (median age 65 years, range 32–76 years). Four out of five patients were seropositive for antibodies directed to anti-Sjögren's syndrome related antigen A (anti-SSA). These four were also seropositive for RF. Biopsies were snap-frozen in liquid nitrogen and cryopreserved at −80°C until use. The biopsies were without MALT lymphoma and had a Chisholm score between 2 and 4. In parotid gland biopsies of four patients LELs were present in the striated ducts. Usage of these biopsies for research purposes was with approval from the medical ethical committee of the University Medical Center of Groningen, Groningen, the Netherlands (METc2013.066). All pSS patients provided informed consent.

**Table 1:** Characteristics of pSS patients.

**Patient biopsy**	**Gender M/F**	**Age at biopsy**	**SWS ml/min**	**RF**	**ANA**	**Anti SSA**	**Anti SSB**	**Chisholm score**	**GC**	**LEL**
pSS1	F	65	0.13	+	+	+	+	4	−	+
pSS2	F	67	0.43	+	−	+	−	3	−	+
pSS3	F	71	0.005	+	+	+	+	4	−	+
pSS4	F	38	0.43	−	−	−	−	3	−	-
pSS5	F	32	0.46	+	+	+	x^*^	2	−	+

*SWS, stimulated whole saliva; RF, rheumatoid factor; ANA, antinuclear antibody; SSA, Sjögren's-syndrome-related antigen A; SSB, Sjögren's-syndrome-related antigen B; GC, germinal center; LEL, lymphoepithelial lesion*.

* *x, data not available*.

### Laser Microdissection

Serial parotid gland cryosections of 9 μm thickness, were mounted on glass slides covered with polyethylene naphthalene-membrane (MembraneSlide 1.0 PEN, Carl Zeiss Microscopy GmbH, Germany), fixed in acetone, stained with Mayer's hematoxylin, washed with diethyl pyrocarbonate-treated tap water, dehydrated with alcohol and air dried. Periductal infiltrates and striated ducts (with and without LELs) were microdissected using a LMD6000 laser microdissection system (Leica, Wetzlar, Germany). Ducts were taken from areas of the parotid biopsy containing periductal infiltrates. Multiple microdissected areas from serially cut periductal regions or striated ducts of individual patients were pooled into the adhesive cap of the collection tube (AdhesiveCap500, Carl Zeiss Microscopy GmbH, Germany). After harvesting the microdissected areas, 65 μl lysis buffer (RNeasy Micro Kit; Qiagen, Leusden, The Netherlands) was added and subsequently RNA was isolated. In Supplementary Figure 2 we show an example of microdissected striated ducts and periductal infiltrates.

### RNA Isolation and cDNA Synthesis

Total RNA was extracted using the RNeasy Micro kit (Qiagen, Leusden, The Netherlands), according to the manufacturer's protocol. cDNA was subsequently synthesized using Superscript RT-III in a total volume of 20 μl containing 200 ng of random hexamer primers (Invitrogen, Carlsbad, CA, USA), 20 U RNase inhibitor (Invitrogen), and 0.5 mM dNTP (Invitrogen).

### IGHV Gene Amplification and Cloning

Linear amplification of IGHV genes was based upon the multiplex PCR of the Biomed-2 collaborative study (27) using VH (1–6)-FR1 primers and the JH consensus primer (Supplementary Table 1). cDNA was amplified in a total volume of 50 μl Taq buffer (with KCl) containing 10 pmol of each primer (Biolegio BV, Nijmegen, The Netherlands), 2 mM of each dNTP, 1.5 mM Mg_2_Cl and 0.5 U Taq DNA Polymerase (Invitrogen). PCR products were gel-purified (PureLink^®^ Quick Gel Extraction Kit (Invitrogen) before cloning into the pCR™4-TOPO vector (TOPO^®^TA Cloning^®^Kit for Sequencing, Invitrogen). The recombinant vector was transformed into chemically competent E. coli (One Shot™ TOP10, Invitrogen) and grown in kanamycin selective LB medium. For each transformation 50 colonies were randomly picked. Plasmids were then isolated with the Purelink™96 Plasmid Purification System, according to manufacturer's protocol. Finally, IGHV transcripts were sequenced (Macrogen Europe, Amsterdam, The Netherlands).

### Analysis of Rearranged Immunoglobulin Genes

IGHV sequences were analyzed as described before (24). Briefly, sequences were aligned with the human Ig set of the IMGT reference directory (28). Only productive sequences, without insertions and deletions, were included in this study. One hundred percent identical IGHV sequences obtained from a single biopsy were counted as one. Sequences with one or more nucleotide difference were considered as unique sequences. IGHV sequences using the same IGHV germline gene with presence of shared mutations and (near-)identical amino acid sequence at the CDR3 region, were considered clonally related.

### RF Sequence Analysis

It has been shown that in ~40 % of the salivary gland MALT lymphomas cases, high-affinity stereotypic rheumatoid factor (RF) B-cell receptors (BCR) have been found. The VH-CDR3 amino acid sequences of the BCRs show strong homology to the VH-CDR amino acid sequence of RFs (19). Five groups of stereotypic RFs have been described from which the VH-CDR3 have been encoded by specific VDJ rearrangements. Namely two different IGHV1-69/JH4 (V1-69-RF and WOL-RF) rearrangements, IGHV3-7/JH3, IGHV4-59/JH2, and IGHV4-59/JH5 rearrangements. The amino acid of the CDR3 of those RF sequences is shown in Supplementary Table 3 where they are compared to the homologous sequences from the pSS patients. Because the presence of RF in serum of pSS patient was found to be an independent predictor of non-Hodgkin's Lymphoma, including MALT lymphoma (29), we examined the presence of RF encoding IGHV sequences in microdissected parotid gland areas. We therefore compared the VH-CDR3 amino acid sequence of our IGHV sequences for homology with VH-CDR3 amino acid sequence of known RFs, using the NCBI Protein-BLAST algorithm (blastp; search for short nearly exact matches) (19, 22). VH-CDR3 amino acids were considered homologs if sharing at least 60% sequence homology and a length difference between the CDR3 sequences of 3 amino acids or less (24).

### Gene Expression Analysis by Quantitative RT-PCR

Gene transcript expression levels of CD20 and FcRL4 (CD307d) were measured with quantitative reverse transcription PCR (RT-qPCR) using the CFX386 Real-Time PCR detection system and reagents from BioRad (BioRad, Lunteren, The Netherlands). ß-actin was used as housekeeping gene. In short, cDNA and 150 nM primers (Supplementary Table 1) were diluted in iQ™SYBR^®^Green supermix to a final solution of 10 μl, according to the manufacturer's protocol. Each reaction was performed in triplicate. A two-step cycling protocol was used consisting of an initial denaturation/hot start step at 95°C for 30 s, followed by 40 repetitive cycles of denaturing at 95°C for 15 s, annealing, and extension at 60°C for 30 s. Finally, a meltdown of the product from 60 to 95°C with an increment of 5°C every 5 s was performed. The CFX manager software was used to analyze the data. The relative gene expression of CD20 and FcRL4/CD307d was calculated using the double delta Ct analysis (30).

### Statistical Analysis

All statistical analyses were performed using GraphPad Prism software (version 8.0). Statistical comparisons of data from ductal and periductal derived IGHV sequences were carried out using Wilcoxon matched-pairs signed rank test. *P* < 0.05 were considered as statistical significant.

## Results

The total surface of the microdissected areas per patient ranged from 28 to 54 μm^2^ for striated ducts and 23 to 56 μm^2^ for periductal infiltrates. Virtually all B-cells in the striated ducts express FcRL4, whereas the number of FcRL4^+^ B-cells in the periductal areas is much lower (15). To confirm that FcRL4^+^ B-cells are indeed strongly enriched in the microdissected striated ducts, we performed RT-qPCR for relative levels of FcRL4 transcripts. mRNA transcripts from ducts and infiltrate were amplified for both CD20 and FcRL4 and quantified using the double delta Ct method. By calculating the ratio FcRL4/CD20 gene expression, we found up to 5-fold more FcRL4 expression in the striated ducts compared to the periductal infiltrates (Supplementary Figure 1).

### VH-Gene Family Usage of Intraductal B-Cells Is Similar to That of Periductal B-Cells

**Figure 1:**
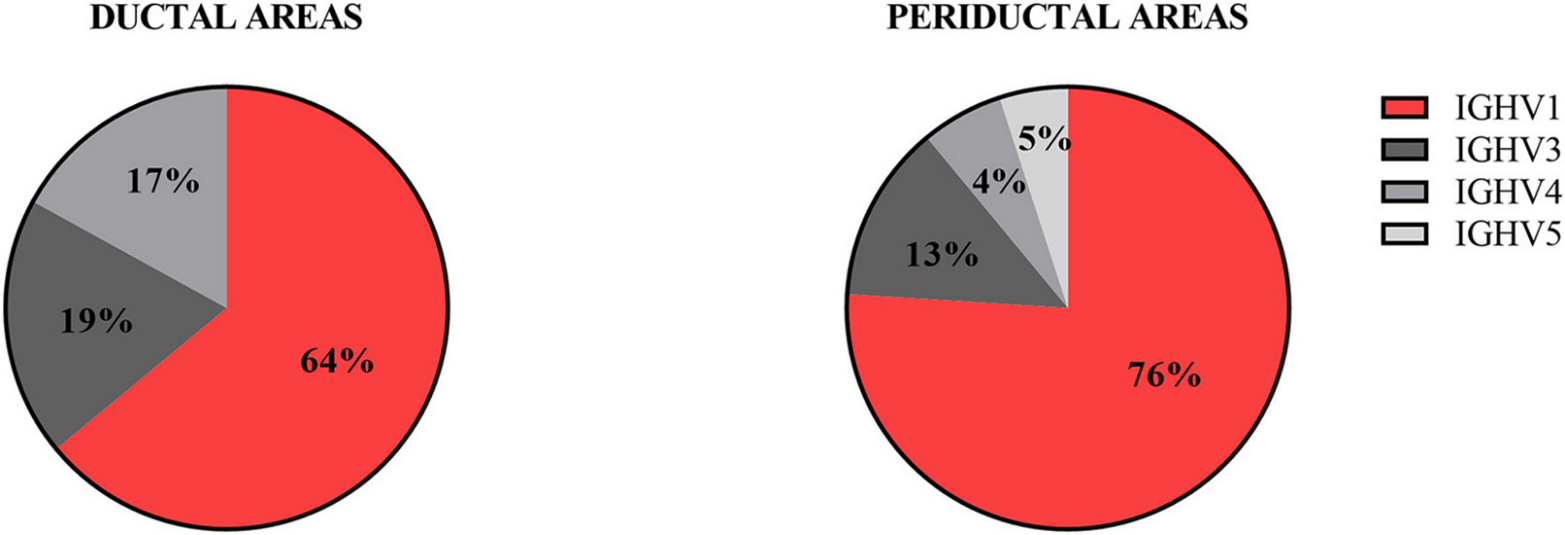
Frequency of VH-gene usage by intraductal and periductal B-cells. The IGHV genes of B-cells present in microdissected areas were sequenced and analyzed by IMGT/V-QUEST for VH-gene repertoire usage.

**Figure 2:**
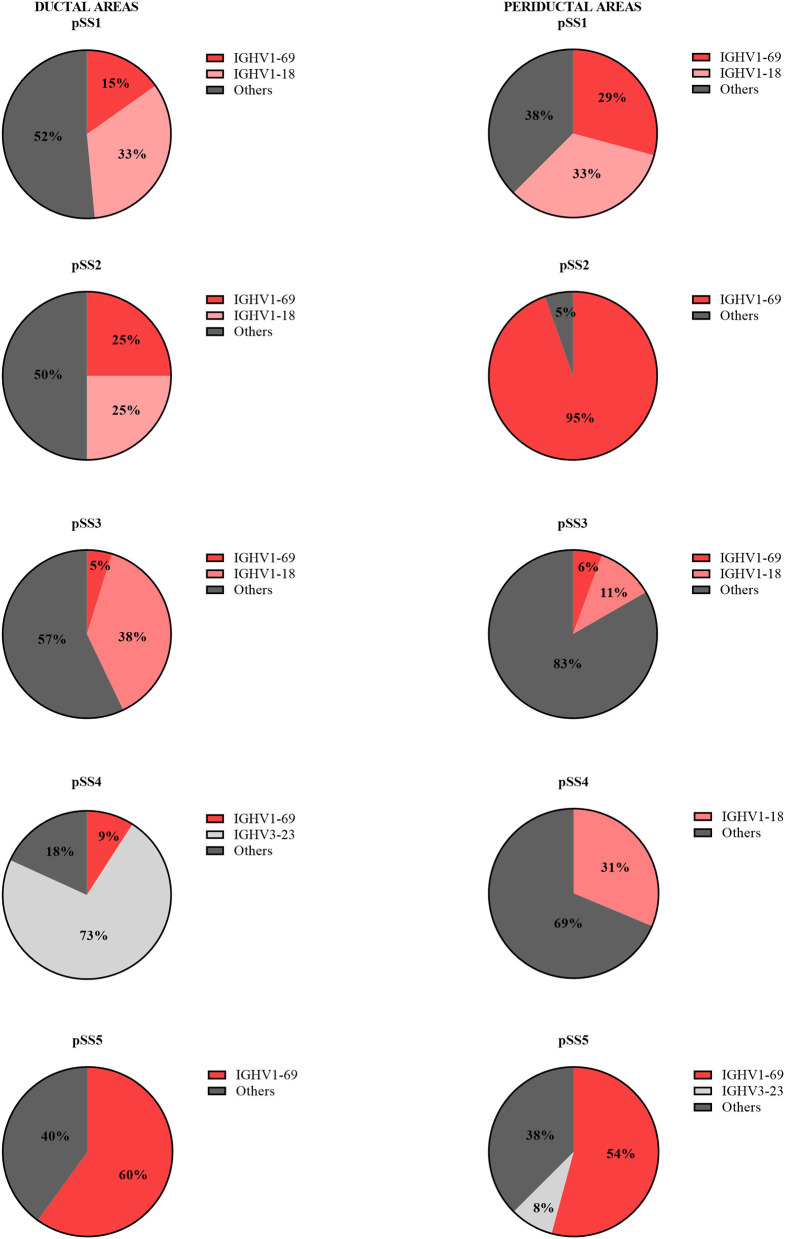
Frequency of VH-gene usage by intraductal and periductal B-cells per pSS patient. The IGHV genes of B-cells present in microdissected areas were sequenced and analyzed by IMGT/V-QUEST for VH-gene repertoire usage.

Since the number of B-cells within the microdissected areas, in particular in striated ducts, is relatively low, we analyzed IGHV genes after cloning IGHV transcripts into appropriate vectors, rather than by deep sequencing. A total of 214 unique IGHV sequences was collected from microdissected areas of five pSS parotid biopsies. Of these sequences, 96 unique intraductal IGHV sequences were obtained from microdissected striated ducts (15–33 IGHV sequences per patient), and 118 unique periductal IGHV sequences from microdissected periductal infiltrates (16–37 IGHV sequences per patient). IGHV sequences from both microdissected ducts and infiltrates represented most of the VH-gene families. The majority of IGHV genes derived from both the microdissected striated ducts and periductal infiltrates were encoded by VH1 genes (64 and 76%, respectively), followed by VH3 genes (19 and 13%), and VH4 genes (17 and 6%). No other IGHV gene families were used by B-cells within the striated ducts, whereas within the periductal infiltrates, 5% of the IGHV genes were encoded by VH5 family genes (Figure 1, Supplementary Table 2). There were a few dominant IGVH-genes present in both ductal and periductal derived IGHV sequences. In both regions, IGHV1-69 and IGHV1-18 were most abundantly used (Table 2, Figure 2 and Supplementary Table 2). Although the usage of IGHV1-69 seems to be 2-fold higher in periducts, this is most likely due to a large VH1-69 clone in pSS2 that comprises 95% of all periductal IGHV sequences from this patient (Table 3). Among the VH3-genes, IGHV3-23 was most frequently present in the striated ducts, especially in pSS4. In this patient, more than 90% of the intraductal IGHV sequences were encoded by VH3 family genes. In pSS4 particularly VH3-23 was strongly (~73%) overrepresented in the striated ducts compared with all other ductal and periductal derived IGHV sequences. Of note, this patient did not exhibit LELs in the parotid salivary gland, which may possibly account for a relative low number of IGHV sequences collected from the striated ducts and possibly also a different intraductal IGHV gene repertoire, compared to the other patients (12).

**Table 2:** Frequency of VH-gene usage of all IGHV sequences derived from ductal and periductal areas.

**IGHV genes**	**Ductal areas (%)**	**Periductal areas (%)**
VH1-2	8.3	7.6
VH1-3	1.0	0
VH1-8	10.4	8.4
VH1-18	24.0	12.6
VH1-69	20.8	47.1
VH1-46	0	0.8
VH3-7	0	0.8
VH3-9	0	0.8
VH3-15	0	1.7
VH3-21	3.1	0
VH3-23	8.3	1.7
VH3-33	1.0	0.8
VH3-39	0	4.2
VH3-48	3.1	0
VH3-49	1.0	0.8
VH3-73	0	0.8
VH3-74	2.1	0.8
VH4-4	0	0.8
VH4-38	1.0	0
VH4-39	3.1	0
VH4-49	6.2	1.7
VH4-59	6.2	2.5
VH4-61	0	0.8
VH5-10	0	1.7
VH5-51	0	3.4

**Table 3:** Summary of characteristics of clones found in microdissected striated ducts and periductal infiltrates from parotid salivary gland biopsies per pSS patient.

**Patient**	**Clone**	**VH-gene**	**JH-gene**	**Origin of IGHV sequence (number of clonally related sequences)**	
				**Ductal areas**	**Periductal areas**
pSS1	1	IGHV1-18	IGHJ4	5	
pSS1	2	IGHV1-69	IGHJ5	2	
pSS1	3	IGHV1-8	IGHJ4	2	1
pSS1	4	IGHV1-18	IGHJ4		4
pSS1	5	IGHV1-2	IGHJ6		3
pSS1	6	IGHV1-69	IGHJ2		2
pSS1	7	IGHV5-51	IGHJ2		2
pSS2	8	IGHV1-18	IGHJ5	3	
pSS2	9	IGHV1-69	IGHJ3	4	34
pSS2	10	IGHV4-59	IGHJ6	6	
pSS3	11	IGHV1-18	IGHJ6	5	
pSS3	12	IGHV1-18	IGHJ6	2	
pSS3	13	IGHV1-8	IGHJ4	5	2
pSS3	14	IGHV4-59	IGHJ5	5	1
pSS3	15	IGHV1-8	IGHJ4		3
pSS3	16	IGHV1-2	IGHJ4		2
pSS4	17	IGHV3-21	IGHJ4	2	
pSS4	18	IGHV3-23	IGHJ5	6	
pSS4	19	IGHV3-23	IGHJ4	2	
pSS4	20	IGHV1-18	IGHJ5		2
pSS4	21	IGHV3-15	IGHJ4		2
pSS4	22	IGHV4-39	IGHJ3		2
pSS4	23	IGHV4-39	IGHJ4		2
pSS5	24	IGHV1-2	IGHJ3	2	
pSS5	25	IGHV1-2	IGHJ4	2	
pSS5	26	IGHV1-69	IGHJ4	7	
pSS5	27^*^	IGHV1-69	IGHJ4	1	4
pSS5	28	IGHV1-69	IGHJ4		2
pSS5	29	IGHV1-69	IGHJ4		2
pSS5	30	IGHV1-69	IGHJ4		2
pSS5	31	IGHV1-69	IGHJ6		3
pSS5	32	IGHV1-8	IGHJ6		2

* *Clone with IGHV sequences with CDR3-RF homology*.

### The Vast Majority of Intraductal and Periductal B-Cells Carry Mutated IGHV Genes

IGHV sequences with a VH-gene ≤ 1 nucleotide difference to the nearest reference VH germline gene were considered as germline sequences. Mutational analysis of the IGHV transcripts derived from both intraductal and periductal B-cells present in parotid gland biopsies, revealed only a very low (<10%) number of germline sequences. In the pool of IGHV sequences derived from the ducts, we observed five out of 96 germline sequences (5%) (Supplementary Table 2). All these germline sequences originated from one patient (pSS1). In the pool of IGHV sequences derived from the periductal infiltrate, we observed a similar low proportion of germline sequences: 10 out of 118 sequences (8%) were in germline configuration. These germline sequences originated from 3 pSS patients (pSS1, pSS2, and pSS4) (Supplementary Table 2). The low frequency of germline encoded IGHV transcripts reflects the presence of relatively low numbers of naïve B-cells and high numbers of memory (mutated) B-cells, in parotid gland tissue of pSS patients (24).

**Figure 3:**
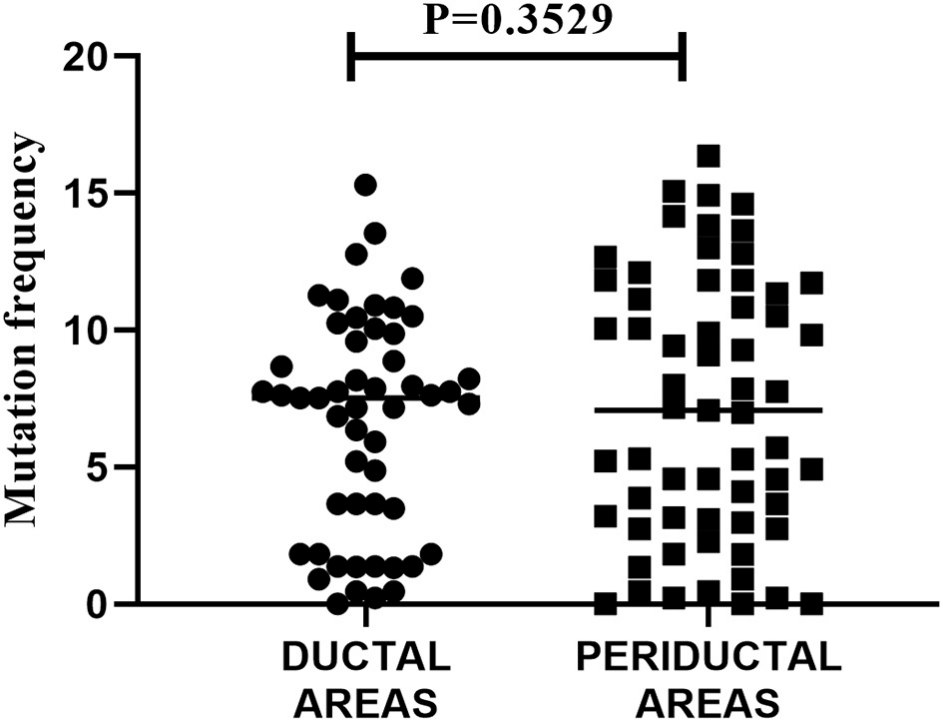
Mutation frequency of all IGHV sequences in striated ducts compared to periductal infiltrates. The nucleotide sequences of the IGHV sequences were aligned to the closest VH-REGION alleles from the IMGT/V-QUEST reference directory sets. The mutation frequency was calculated as 100% minus the % of nucleotide identity. Each dot or square represents an IGHV sequence, the horizontal bars show the median.

Next, we analyzed the mutation frequency of the mutated IGHV sequences (>1 nucleotide difference to closest reference germline gene). To examine whether there is a difference in mutation incidence between intraductal and periductal B-cells, the mutation frequencies of all ductal derived IGHV sequences were compared to all periductal derived IGHV sequences. To prevent bias due to expanded clones, clonally related IGHV sequences were counted as one. For this assessment, the mean mutation frequency of all members of a particular clone were taken. As we show in Figure 3, there was no significant difference in mutation frequency observed in intraductal derived IGHV sequences compared to periductal derived IGHV sequences (*P* = 0.3529).

### Both Striated Ducts and Periductal Areas Comprise B-Cell Clones

To examine the presence of clonal expansions among B-cells located in the striated ducts and/or periductal areas, and to find possible relationships between B-cells at these two sites, we searched for the presence of clonally related IGHV sequences in the microdissected areas. IGHV sequences were defined clonally related when they use the same VH-gene, share mutations within the VH-gene and share near-identical amino acid sequences encoded by the H-CDR3 region. Despite the relative low number of IGHV sequences obtained per patient we were able to detect many clonally related sequences both derived from intraductal and periductal B-cells. Clonally related sequences were observed in all patients (Table 3). In total 32 clones were found. Of all sequences analyzed, 138 sequences (i.e., 57%) belonged to a clone. Out of 32 clones, 28 clones shared identical VH-CDR3 amino acid sequences and four shared near-identical VH-CDR3 regions with up to 4 amino acid difference from each other (Supplementary Table 2). Of these 32 clones, 12 clones were observed among sequences derived from the microdissected ducts and 15 clones from the periductal infiltrates. Interestingly, five clones were composed of sequences derived from microdissected ducts and periductal infiltrates. These shared clones were found in parotid gland tissue of all pSS patients, except for pSS4. Most periductal sequences from patient pSS2 belonged to one large clone. Although this observation might be suggestive for MALT lymphoma, this patient was not diagnosed histopathologically with such a lymphoma. Recent visit to our clinic also showed no indication for MALT development.

VH-gene analysis of all clones showed that most clones (~73%) were of VH1 origin followed by VH3 (12%), VH4 (12%), and VH5 (3%) (Table 3), which roughly reflects the proportion of the various VH-gene families expressed in the biopsies. Accordingly, most of the clones expressing VH1 sequences were encoded by the IGHV1-69 gene (38% of total number of VH1 clones) followed by IGHV1-18 (29%), IGHV1-8 (17%), and IGHV1-2 (17%).

### B-Cell Receptors of Intraductal B-Cells and Periductal B-Cells Occasionally Express RF

Since MALT lymphomas may arise from B-cells associated with striated ducts (15) and neoplastic MALT B-cells frequently express RF (19), we analyzed whether non-malignant intraductal and/or periductal B-cells are enriched in IGHV genes encoding for immunoglobulins with RF activity. We therefore compared VH-CDR3 amino acid sequences of all IGHV sequences for CDR3-RF homology (Supplementary Table 3). Of note, all patients, except for pSS4, were tested seropositive for RF (Table 1). IGHV sequences with VH-CDR3 regions showing homology for CDR3-RF regions were present among IGHV sequences from four out of five patients. In line with the serum data, no RF sequences were observed in patient 4. Only two out of 96 IGHV sequences (2%) derived from B-cells located in the striated duct and 10 out of 118 IGHV sequences (8%) in the periductal infiltrate were homologs to CDR3-RF. Remarkedly, 11 (one intraductal and 10 periductal) IGHV sequences with CDR3-RF homology, were encoded by VH/JH rearrangements characteristic for stereotypic RFs (19, 22) (Supplementary Table 3).

Among the total of 12 IGHV sequences with CDR3-RF homology, 9 (75%) sequences were derived from the parotid biopsy of only one patient, pSS5. These sequences were part of three clones with respectively 5, 2, and 2 members. When the RF sequences that originate from one clone are counted as one, the frequency of RF sequences in the periductal infiltrate becomes 5/118 (4%). Of the largest clone one member was of ductal origin and the remaining four were from the periductal region. The members of the other two clones were all from periductal origin (Table 2). Together, the data indicate that intraductal B-cells are not enriched in B-cells expressing a BCR exhibiting RF specificity.

## Discussion

B-cell hyperactivity is a hallmark of pSS, which may culminate in the formation of MALT lymphomas, which frequently develop within the parotid salivary gland. B-cells located within the striated ducts may be the cells from which the neoplastic B-cells arise (15). These intraductal B-cells are also held responsible for the formation of lymphoepithelial lesions (12), which are critically involved in MALT lymphomagenesis. MALT lymphoma B-cells in pSS parotid glands frequently exhibit RF specificity (19, 20). For this reason we explored whether intraductal B-cells are selected on the basis of specificity of the BCR. Here we observed that there are no major differences in the IGHV gene repertoire of intraductal B-cells and of periductal B-cells. At both locations the vast majority (>90%) of B-cells express mutated IGHV genes, indicating that nearly all cells represent memory cells. Importantly, B-cells expressing VH-CDR3 regions, encoding for RF specificity, are not enriched in striated ducts. An important finding was that a large fraction (57%) of IGHV sequences were clonally related to each other. These sequences were derived from B-cell clones with members either only in the ducts or only in the periductal areas, and importantly, also from clones with members in the ducts and in the periductal areas. Taken together, the data indicate that there is a significant exchange of B-cells located in the striated ducts and B-cells in the periductal region and that recruitment of B-cells into the ducts is most likely not driven by their BCR specificity.

Nearly all, if not all, B-cells located in the striated ducts express FcRL4 (15). Transcriptomic analysis and phenotypic characterization of tonsillar FcRL4^+^ B-cells and FcRL4^+^ B-cells in synovial tissue of patients with rheumatoid arthritis indicate that FcRL4^+^ B-cells are pro-inflammatory, activated, memory cells (31, 32). We recently confirmed this also for FcRL4^+^ B-cells in parotid salivary glands of pSS patients (16). Parotid FcRL4^+^ B-cells of pSS patients show gene expression profile with similarities to chronically activated CD11c^+^T-bet^+^ memory B-cells which are involved in the pathogenesis of systemic lupus erythematosus (18, 33). These findings also revealed that these glandular FcRL4^+^ B-cells express the chemokine receptor CXCR3. CXCR3 can bind to the interferon-induced chemokine CXCL10/IP-10, which is produced in large quantities by ductal epithelial cells in the salivary glands of pSS patients (34). Any activated B-cell, expressing FcRL4 and CXCR3, located in the periductal area can likely be attracted to migrate into the striated ducts by CXCL10, irrespective of the antigen specificity of the B-cell. Once arrived in the striated ducts the (already) activated B-cells may be activated further, possibly supported by cytokines secreted by ductal epithelial cells. The ducts in the pSS salivary glands secrete a wide variety of cytokines that support B-cell activation (35). These cytokines include BAFF and APRIL which can bind to their receptor TACI. This receptor is upregulated in FcRL4^+^ B-cells, and binding of BAFF/APRIL results in enhanced NF-kB mediated B-cell activation (36, 37). Indeed, FcRL4^+^ intraductal B-cells proliferate highly and ~15–20% of the cells in labial and parotid salivary glands of pSS patients are actively dividing as witnessed by their expression of Ki-67 (15). This expansion is also mirrored by the presence of intraductal B-cell clones, which we identified based on IGHV gene analysis. Clonal expansion is, however, clearly not restricted to the ducts, as periductal B-cell clones are also seen. During B-cell activation and clonal expansion some intraductal (activated) B-cells may leave again the striated ducts toward the periductal areas. The intimate relationship between B-cells located within the striated ducts and B-cells present in the periductal infiltrates is underscored by the presence of B-cell clones, with members located at both sites. Since most MALT lymphomas consist of IgM producing cells (19, 20), we have confirmed the presence of IgM, IgG, and IgA producing B-cells in the ductal areas (Supplementary Figure 2). We could confirm this further by analyzing FcRL4+ B-cells sorted from parotid glands by RNA-seq. We found transcripts for IgM as well as IgG and IgA (data not shown).

During humoral immune responses, somatic hypermutation of V-genes associated with selection of memory cells with high affinity BCRs, usually takes place within the germinal centers. Cumulating evidence, however, shows this somatic hypermutation process can also occur outside germinal centers, in particular in autoimmune disease (8, 24, 38, 39). In the salivary gland biopsies of the pSS patients, there was no evidence for the presence of ectopic germinal centers in the tertiary lymphoid tissue, although we cannot formally rule out the possibility that elsewhere in the parotid gland germinal centers might be present. The observed local clonal expansions of both intraductal and periductal B-cells associated with the accumulation of somatic mutations, thus most probably occur independent from germinal centers. FcRL4^+^ B-cells, including FcRL4^+^ B-cells within the salivary glands of pSS patient, have the potency to express activation-induced-deaminase (AID), an enzyme critically involved in somatic hypermutation (15, 16, 31, 40, 41). The proliferative activity, expression of AID, and the association with lymphoepithelial lesions, characteristic for MALT lymphomas, may make the intraductal B-cells prone for neoplastic derailment.

Presence of RF in serum of pSS patients is an independent predictor for (MALT) lymphoma development (29). The majority, if not all MALT lymphomas in salivary glands of pSS patients are composed of B-cells with a BCR with RF specificity (19, 21). These BCRs are mostly encoded by stereotypic CDR3-RF sequences (19, 20). RF sequences were only found in those patients that expressed RF in the serum. We observed that only 2% of the intraductal B-cells express RF- BCRs compared to 4–8% of the periductal B-cells, depending on whether you take the clones into consideration. The higher frequency of CDR3-RFs in the periductal areas was mainly due to presence of three RF-clones in the parotid biopsy of pSS5. Interestingly, one of the B-cell clones with members in the striated ducts and in the periductal region exhibited RF reactivity by expressing a BCR encoded by VH-CDR3 with homology for the stereotypic IGHV1-69-RF found in salivary gland MALT lymphoma as described by Miklos et al. (42). During follow- up of this patient for 9 years, this patient has not developed a MALT lymphoma. Importantly, intraductal B-cells, as well as periductal B-cells, show a low frequency of B-cells with RF-specificity. Also, most of the clones did not express BCRs with homology for (stereotypic) RF sequences, indicating that clonal expansion is not dependent of a BCR with RF specificity. Since more than 40% of the pSS associated glandular MALT lymphomas express RF, expression of RF by a B-cells is apparently an important risk factor for neoplastic transformation of B-cells toward MALT lymphoma. B-cells expressing BCRs with RF specificity may be extra stimulated in an environment with immune complexes binding to the RF and nucleoprotein autoantigens (SSA and SSB) binding to e.g., toll-like receptors. The stimulatory environment of the striated ducts as a source of both B-cell activating cytokines and SSA/B autoantigens may contribute to the preferential activation and proliferation of the intraductal B-cells once attracted to the striated ducts. The expression of AID by FcRL4^+^ intraductal B-cells may further contribute in derailment toward MALT lymphomas, by mutating non-immunoglobulin genes (43). Further support for this hypothesis comes from a recent study of Singh et al. (44). They showed that in pSS patients, pathogenic RF-expressing B-cells are formed exclusively in RF-expressing cells after acquiring lymphoma driver mutations. Apparently, RF-specificity is required for the pathogenic and neoplastic transformation of the cells.

To conclude, it is unlikely that RF specific B-cells are preferentially recruited from the periductal regions into the striated ducts in the salivary glands of patients with pSS. Instead, we postulate that in principle any activated B-cell can migrate into the striated ducts, irrespective of its BCR specificity. The high frequency of MALT lymphomas expressing BCRs with RF-specificity suggests that this may be an important risk factor toward MALT lymphoma development, in particular in the stimulatory microenvironment of the striated duct.

## Data Availability Statement

This article contains previously unpublished data. GenBank Accession Numbers are MT151387–MT151600.

## Ethics Statement

The studies involving human participants were reviewed and approved by the Medical Ethical Committee of the University Medical Center of Groningen, Groningen, The Netherlands (METc2013.066). The patients/participants provided their written informed consent to participate in this study.

## Author Contributions

FK, HB, and AVisse conceived and designed the study. HB and AVissi recruited the patients and provided clinical data. AVisse performed the assays, acquired and analyzed data, and wrote the concept of the manuscript. FK, GV, BV, AVissi, HB, and NB were involved in data analysis, contributed to the discussion, and editing of the manuscript. RB was involved in the analysis of the RF sequences. All authors contributed to the article and approved the submitted version.

## Conflict of Interest

The authors declare that the research was conducted in the absence of any commercial or financial relationships that could be construed as a potential conflict of interest.
