# Supplementary material for: Repertoire Analysis of B-Cells Located in Striated Ducts of Salivary Glands of Patients With Sjögren's Syndrome
Source: Front Immunol. 2020 Jul 14;11:1486. doi: 10.3389/fimmu.2020.01486 (PMC7372116; doi:10.3389/fimmu.2020.01486)
Supplement: Supplementary file 1 [file Table_1.DOCX]

| PRIMER | SEQUENCE 5’ – 3’ |
| --- | --- |
| CD20-F | GCACCCATCTGTGTGACTGT |
| CD20-B | ATGGCAGCAAAGAGGCTC AA |
| FcRL4-F | GGGCGTCCT TGCTGGCCT TT |
| FcRL4-B | GGGTGTTTCCTGGGGTCAGGG T |
| VH1-F | GGCCTCAGTGAAGGTCTCCTGCAAG |
| VH2-F | GTCTGGTCCTACGCTGGTGAAACCC |
| VH3-F | CTGGGGGGTCCCTGAGACTCTCCTG |
| VH4-F | CTTCGGAGACCCTGTCCCTCACCTG |
| VH5-F | CGGGGAGTCTCTGAAGATCTCCTGT |
| VH6-F | TCGCAGACCCTCTCACTCACCTGTG |
| JH-B | CTTACCTGAGGAGACGGTGACC |
| ß-actin-F | GAGCGGGAAATCGTGCGTGAC |
| ß-actin-B | AGGAAGGAAGGCTGGAAGAGTGC |

Supplementary Table 1. Primer sequences used in PCR.
