# Supplementary material for: Repertoire Analysis of B-Cells Located in Striated Ducts of Salivary Glands of Patients With Sjögren's Syndrome
Source: Front Immunol. 2020 Jul 14;11:1486. doi: 10.3389/fimmu.2020.01486 (PMC7372116; doi:10.3389/fimmu.2020.01486)
Supplement: Supplementary file 2 [file Table_2.DOCX]

| **PATIENT** | **No** | **AREA** | **VH-GENE and allele** | **VH-GENE identity (nt)** | **VH-GENE mutation (%)** | **JH-GENE and allele** | **DH-GENE and allele** | **AA JUNCTION** |
| --- | --- | --- | --- | --- | --- | --- | --- | --- |
| pSS1 | 1 | Ductal | IGHV1-18*01 F | 204/219 nt | 6.85 | IGHJ5*02 F | IGHD4-11*01 ORF | CARAHFTNNGDWLDPW |
| pSS1 | 2 | Ductal | IGHV1-18*01 F | 208/219 nt | 5.02 | IGHJ4*02 F | IGHD2-15*01 F | CARDLVVGAAPGASGYW |
| pSS1 | 3 | Ductal | IGHV1-18*01 F | 208/219 nt | 5.02 | IGHJ4*02 F | IGHD1-26*01 F | CARDLVVGAAPGASGYW |
| pSS1 | 4 | Ductal | IGHV1-18*01 F | 210/219 nt | 4.11 | IGHJ4*02 F | IGHD1-26*01 F | CARDLVVGAAPGASGYW |
| pSS1 | 5 | Ductal | IGHV1-18*01 F | 207/219 nt | 5.48 | IGHJ4*02 F | IGHD1-26*01 F | CARDLVVGAAPGASGYW |
| pSS1 | 6 | Ductal | IGHV1-18*01 F | 205/219 nt | 6.39 | IGHJ4*03 F | IGHD1-26*01 F | CARDLVVGAAPGASGYW |
| pSS1 | 7 | Ductal | IGHV1-18*01 F | 218/219 nt | 0.46 | IGHJ4*02 F | IGHD6-19*01 F | CARDWGSSGWPAYW |
| pSS1 | 8 | Ductal | IGHV1-18*01 F | 211/219 nt | 3.65 | IGHJ6*02 F | IGHD3-9*01 F | CARENPAYNISTGYLTSYYGMDVW |
| pSS1 | 9 | Ductal | IGHV1-18*01 F | 198/219 nt | 9.59 | IGHJ4*02 F | IGHD3-22*01 F | CARFYDDSRYYLGFDYW |
| pSS1 | 10 | Ductal | IGHV1-18*01 F | 197/219 nt | 10.05 | IGHJ6*02 F | IGHD1-26*01 F | CARGRGVGPTPSGNHYFAVELW |
| pSS1 | 11 | Ductal | IGHV1-18*01 F | 215/219 nt | 1.83 | IGHJ3*02 F | IGHD4-23*01 ORF | CARSVGGRVVNQGDAFDIW |
| pSS1 | 12 | Ductal | IGHV1-2*02 F | 201/219 nt | 8.22 | IGHJ5*02 F | IGHD3-9*01 F | CARDPSGDVLSVNGYKRLNWFDPW |
| pSS1 | 13 | Ductal | IGHV1-2*02 F | 202/219 nt | 7.76 | IGHJ4*02 F | IGHD3-3*01 F | CARAQLTIFGVVILDYW |
| pSS1 | 14 | Ductal | IGHV1-2*02 F | 196/219 nt | 10.50 | IGHJ5*02 F | IGHD1-1*01 F | CARDSGATYDWFEPW |
| pSS1 | 15 | Ductal | IGHV1-2*04 F | 211/219 nt | 3.65 | IGHJ6*02 F | IGHD4-17*01 F | CARGDYRDSGAGYYYGMDVW |
| pSS1 | 16 | Ductal | IGHV1-3*01 F | 211/219 nt | 3.65 | IGHJ4*02 F | IGHD5-12*01 F | CARDRSGYVATLLDYW |
| pSS1 | 17 | Ductal | IGHV1-69*01 F | 201/219 nt | 8.22 | IGHJ5*02 F | IGHD1-26*01 F | CARSDSGSYYDFTFDRW |
| pSS1 | 18 | Ductal | IGHV1-69*01 F | 199/219 nt | 9.13 | IGHJ5*02 F | IGHD1-26*01 F | CARSDSGSYYDFTFDRW |
| pSS1 | 19 | Ductal | IGHV1-69*01 F | 216/219 nt | 1.37 | IGHJ5*02 F | IGHD3-22*01 F | CARDTSRPYYDSSGGGFDPW |
| pSS1 | 20 | Ductal | IGHV1-69*01 F | 215/219 nt | 1.83 | IGHJ3*02 F | IGHD5-24*01 ORF | CARPEMATKTAFDIW |
| pSS1 | 21 | Ductal | IGHV1-69*06 F | 203/219 nt | 7.31 | IGHJ5*02 F | IGHD2-2*01 F | CARDSASVGHPPAGMAWFDAW |
| pSS1 | 22 | Ductal | IGHV1-8*01 F | 216/219 nt | 1.37 | IGHJ6*02 F | IGHD3-3*01 F | CAEDFWSGQTPPMDVW |
| pSS1 | 23 | Ductal | IGHV1-8*01 F | 216/219 nt | 1.37 | IGHJ5*02 F | IGHD3-22*01 F | CARGPSGHYDPSVWGTWFDPW |
| pSS1 | 24 | Ductal | IGHV1-8*01 F | 219/219 nt | 0.00 | IGHJ5*02 F | IGHD4-23*01 ORF | CAREMWELW |
| pSS1 | 25 | Ductal | IGHV1-8*01 F | 219/219 nt | 0.00 | IGHJ4*02 F | IGHD6-13*01 F | CARGGWEGYSSSWGDYW |
| pSS1 | 26 | Ductal | IGHV1-8*01 F | 218/219 nt | 0.46 | IGHJ4*02 F | IGHD6-13*01 F | CARGGWEGYSSSWGDYW |
| pSS1 | 27 | Ductal | IGHV3-21*01 F | 206/223 nt | 7.62 | IGHJ5*01 F | IGHD2-2*01 F | CARGPMLSQLLSDGGSGWFDSW |
| pSS1 | 28 | Ductal | IGHV3-33*01 F | 206/223 nt | 7.62 | IGHJ6*02 F | IGHD3-16*01 F | CARERLGGSGKRYGMDVW |
| pSS1 | 29 | Ductal | IGHV3-48*03 F | 221/223 nt | 0.90 | IGHJ4*02 F | IGHD3-16*01 F | CASDGGFLGFDYW |
| pSS1 | 30 | Ductal | IGHV3-48*03 F | 222/223 nt | 0.45 | IGHJ1*01 F | IGHD6-19*01 F | CARDQGSGWYPTTPEYFQHW |
| pSS1 | 31 | Ductal | IGHV3-49*03 F | 221/229 nt | 3.49 | IGHJ5*02 F | IGHD1-7*01 F | CTRVNYNWIYGWFDPW |
| pSS1 | 32 | Ductal | IGHV3-74*03 F | 201/223 nt | 9.87 | IGHJ4*02 F | IGHD5-12*01 F | CARLVASDYW |
| pSS1 | 33 | Ductal | IGHV4-39*01 F | 223/226 nt | 1.33 | IGHJ4*02 F | IGHD5-12*01 F | CARENKYSGYGGVDLYW |
| pSS1 | 34 | Periductal | IGHV1-18*01 F | 214/219 nt | 2.28 | IGHJ4*02 F | IGHD3-22*01 F | CARDIFRGSSGFLMLGFEHW |
| pSS1 | 35 | Periductal | IGHV1-18*01 F | 218/219 nt | 0.46 | IGHJ4*02 F | IGHD3-10*01 F | CARAAPGGSGSYNSYFDSW |
| pSS1 | 36 | Periductal | IGHV1-18*01 F | 211/219 nt | 3.65 | IGHJ4*02 F | IGHD3-10*01 F | CARAAPGGSG*G*YNSYFDSW |
| pSS1 | 37 | Periductal | IGHV1-18*01 F | 212/219 nt | 3.20 | IGHJ4*02 F | IGHD3-10*01 F | CARAAPGGSGSYNSYFDSW |
| pSS1 | 38 | Periductal | IGHV1-69*06 F | 203/219 nt | 8.22 | IGHJ4*02 F | IGHD3-10*01 F | CARAAPGGSGSYNSYFDSW |
| pSS1 | 39 | Periductal | IGHV1-18*01 F | 219/219 nt | 0.00 | IGHJ4*02 F | IGHD3-16*02 F | CARAEMITFGGVIVGGYYFDYW |
| pSS1 | 40 | Periductal | IGHV1-18*01 F | 219/219 nt | 0.00 | IGHJ4*02 F | IGHD6-19*01 F | CASATGSGWDLSFDYW |
| pSS1 | 41 | Periductal | IGHV1-18*01 F | 209/219 nt | 4.57 | IGHJ4*02 F | IGHD3-22*01 F | CARDRPDLYQYDSSGYAQMDYW |
| pSS1 | 42 | Periductal | IGHV1-18*01 F | 211/219 nt | 3.65 | IGHJ4*02 F | IGHD6-19*01 F | CARAPTYSSGWSGYYFDYW |
| pSS1 | 43 | Periductal | IGHV1-2*02 F | 198/219 nt | 9.59 | IGHJ6*02 F | IGHD5-18*01 F | CARGNSPYGMDVW |
| pSS1 | 44 | Periductal | IGHV1-2*02 F | 199/219 nt | 9.13 | IGHJ6*02 F | IGHD5-18*01 F | CARGNSPYGMDVW |
| pSS1 | 45 | Periductal | IGHV1-2*02 F | 199/219 nt | 9.13 | IGHJ6*02 F | IGHD5-18*01 F | CARGNSPYGMDVW |
| pSS1 | 46 | Periductal | IGHV1-2*02 F | 196/219 nt | 10.50 | IGHJ4*02 F | IGHD3-22*01 F | CATDLRYSSSNYW |
| pSS1 | 47 | Periductal | IGHV1-69*01 F | 213/219 nt | 2.74 | IGHJ5*02 F | IGHD6-13*01 F | CARGAAAGREDWFDPW |
| pSS1 | 48 | Periductal | IGHV1-69*01 F | 209/219 nt | 4.57 | IGHJ5*02 F | IGHD1-20*01 F | CARDGVTGTTHNWFDPR |
| pSS1 | 49 | Periductal | IGHV1-69*01 F | 218/219 nt | 0.46 | IGHJ5*02 F | IGHD4-23*01 ORF | CARLDYGGNGLLDPW |
| pSS1 | 50 | Periductal | IGHV1-69*01 F | 201/219 nt | 8.22 | IGHJ6*02 F | IGHD1-26*01 F | CATTGDPVGAIVGWRSSMDVW |
| pSS1 | 51 | Periductal | IGHV1-69*01 F | 202/219 nt | 7.76 | IGHJ6*02 F | IGHD1-26*01 F | CATTGDPVGAIVGWRSSMDVW |
| pSS1 | 52 | Periductal | IGHV1-69*06 F | 215/219 nt | 1.83 | IGHJ6*02 F | IGHD2-15*01 F | CASPDFGVEGSYYYYGMDVW |
| pSS1 | 53 | Periductal | IGHV1-8*01 F | 217/219 nt | 0.91 | IGHJ6*02 F | IGHD3-16*02 F | CAKLTKSYGYNYYYYYGMDVW |
| pSS1 | 54 | Periductal | IGHV1-8*02 F | 212/219 nt | 3.20 | IGHJ4*02 F | IGHD6-13*01 F | CARGGWEGYSSSWGDYW |
| pSS1 | 55 | Periductal | IGHV5-51*01 F | 222/222 nt | 0.00 | IGHJ2*01 F | IGHD3-22*01 F | CARHEGDYYDSSGYSSYWYFDLW |
| pSS1 | 56 | Periductal | IGHV5-51*01 F | 221/222 nt | 0.45 | IGHJ2*01 F | IGHD3-22*01 F | CARHEGDYYDSSGYSSYWYFDLW |
|  |  |  |  |  |  |  |  |  |
| pSS2 | 1 | Ductal | IGHV1-18*01 F | 202/220 nt | 8.18 | IGHJ4*02 F | IGHD3-3*01 F | CARAFTSGYVDYW |
| pSS2 | 2 | Ductal | IGHV1-18*01 F | 196/220 nt | 10.91 | IGHJ5*02 F | IGHD3-16*01 F | CARVQGNYVLDPW |
| pSS2 | 3 | Ductal | IGHV1-18*01 F | 195/219 nt | 10.96 | IGHJ5*02 F | IGHD3-16*01 F | CARVQGNYVLDPW |
| pSS2 | 4 | Ductal | IGHV1-18*01 F | 194/219 nt | 11.42 | IGHJ5*02 F | IGHD3-16*01 F | CARVQGNYVLDPW |
| pSS2 | 5 | Ductal | IGHV1-69*01 F | 210/220 nt | 4.55 | IGHJ3*02 F | IGHD3-3*01 F | CAREIFQPGAFDIW |
| pSS2 | 6 | Ductal | IGHV1-69*01 F | 196/219 nt | 10.50 | IGHJ3*02 F | IGHD3-9*01 F | CAREIFQPGAFDIW |
| pSS2 | 7 | Ductal | IGHV1-69*01 F | 211/220 nt | 4.09 | IGHJ3*02 F | IGHD3-3*01 F | CAREIFQPGAFDIW |
| pSS2 | 8 | Ductal | IGHV1-69*01 F | 210/220 nt | 4.55 | IGHJ3*02 F | IGHD3-9*01 F | CAREIFQPGAFDIW |
| pSS2 | 9 | Ductal | IGHV4-39*01 F | 215/226 nt | 4.87 | IGHJ6*03 F | IGHD6-19*01 F | CDSGITVAGHYYYYYMDVW |
| pSS2 | 10 | Ductal | IGHV4-39*07 F | 209/226 nt | 7.52 | IGHJ6*03 F | IGHD2-21*01 F | CARMWGSGRANYYYYMGVW |
| pSS2 | 11 | Ductal | IGHV4-59*01 F | 202/220 nt | 8.18 | IGHJ6*03 F | IGHD2-21*01 F | CARMWGSGRANYYYYMGVW |
| pSS2 | 12 | Ductal | IGHV4-59*01 F | 203/220 nt | 7.73 | IGHJ6*03 F | IGHD2-21*01 F | CARMWGSGRANYYYYMGVW |
| pSS2 | 13 | Ductal | IGHV4-59*01 F | 203/220 nt | 7.73 | IGHJ6*03 F | IGHD2-21*01 F | CARMWGSGRANYYYYMGVW |
| pSS2 | 14 | Ductal | IGHV4-59*01 F | 202/220 nt | 8.18 | IGHJ6*03 F | IGHD2-21*01 F | CARMWGSGRANYYYYMGVW |
| pSS2 | 15 | Ductal | IGHV4-59*01 F | 203/220 nt | 7.73 | IGHJ6*03 F | IGHD2-21*01 F | CARMWGSGRANYYYYMGVW |
| pSS2 | 16 | Ductal | IGHV4-59*01 F | 203/220 nt | 7.73 | IGHJ6*03 F | IGHD2-21*01 F | CVRMWGSGRANYYYYMGVW |
|  |  |  |  |  |  |  |  |  |
| pSS2 | 17 | Periductal | IGHV1-46*01 F | 219/220 nt | 0.45 | IGHJ6*03 F | IGHD3-16*01 F | CARGSGPFNMDVW |
| pSS2 | 18 | Periductal | IGHV1-69*01 F | 210/220 nt | 4.55 | IGHJ3*02 F | IGHD3-9*01 F | CAREIFQPGAFDIW |
| pSS2 | 19 | Periductal | IGHV1-69*01 F | 207/220 nt | 5.91 | IGHJ3*02 F | IGHD3-9*01 F | CAREIFQPGAFDIW |
| pSS2 | 20 | Periductal | IGHV1-69*01 F | 207/220 nt | 5.91 | IGHJ3*02 F | IGHD3-9*01 F | CAREIFQPGAFDIW |
| pSS2 | 21 | Periductal | IGHV1-69*01 F | 210/220 nt | 4.55 | IGHJ3*02 F | IGHD3-9*01 F | CAREIFQPGAFDIW |
| pSS2 | 22 | Periductal | IGHV1-69*01 F | 208/220 nt | 5.45 | IGHJ3*02 F | IGHD3-9*01 F | CAREIFQPGAFDIW |
| pSS2 | 23 | Periductal | IGHV1-69*01 F | 210/220 nt | 4.55 | IGHJ3*02 F | IGHD3-3*01 F | CAREIFQPGAFDIW |
| pSS2 | 24 | Periductal | IGHV1-69*01 F | 211/220 nt | 4.09 | IGHJ3*02 F | IGHD3-3*01 F | CAREIFQPGAFDIW |
| pSS2 | 25 | Periductal | IGHV1-69*01 F | 202/220 nt | 8.18 | IGHJ3*02 F | IGHD3-3*01 F | CAREIFQPGAFDIW |
| pSS2 | 26 | Periductal | IGHV1-69*01 F | 211/220 nt | 4.09 | IGHJ3*02 F | IGHD3-9*01 F | CAREIFQPGAFDIW |
| pSS2 | 27 | Periductal | IGHV1-69*01 F | 210/220 nt | 4.55 | IGHJ3*02 F | IGHD3-9*01 F | CAREIFQPGAFDIW |
| pSS2 | 28 | Periductal | IGHV1-69*01 F | 212/220 nt | 3.64 | IGHJ3*02 F | IGHD3-9*01 F | CAREIFQPGAFDIW |
| pSS2 | 29 | Periductal | IGHV1-69*01 F | 206/220 nt | 6.36 | IGHJ3*02 F | IGHD3-3*01 F | CAREIFQPGAFDIW |
| pSS2 | 30 | Periductal | IGHV1-69*01 F | 210/220 nt | 4.55 | IGHJ3*02 F | IGHD3-9*01 F | CAREIFQPGAFDIW |
| pSS2 | 31 | Periductal | IGHV1-69*01 F | 210/220 nt | 4.55 | IGHJ3*02 F | IGHD3-9*01 F | CAREIFQPGAFDIW |
| pSS2 | 32 | Periductal | IGHV1-69*01 F | 204/219 nt | 6.85 | IGHJ3*02 F | IGHD3-9*01 F | CAREIFQPGAFDIW |
| pSS2 | 33 | Periductal | IGHV1-69*01 F | 208/219 nt | 5.02 | IGHJ3*02 F | IGHD3-9*01 F | CAREIFQPGAFDIW |
| pSS2 | 34 | Periductal | IGHV1-69*01 F | 209/219 nt | 4.57 | IGHJ3*02 F | IGHD3-3*01 F | CAREIFQPGAFDIW |
| pSS2 | 35 | Periductal | IGHV1-69*01 F | 204/220 nt | 7.27 | IGHJ3*02 F | IGHD3-3*01 F | CAREIFQPGAFDIW |
| pSS2 | 36 | Periductal | IGHV1-69*01 F | 207/220 nt | 5.91 | IGHJ3*02 F | IGHD3-3*01 F | CAREIFQPGAFDIW |
| pSS2 | 37 | Periductal | IGHV1-69*01 F | 212/220 nt | 3.64 | IGHJ3*02 F | IGHD3-3*01 F | CAREIFQPGAFDIW |
| pSS2 | 38 | Periductal | IGHV1-69*01 F | 210/220 nt | 4.55 | IGHJ3*02 F | IGHD3-3*01 F | CAREIFQPGAFDIW |
| pSS2 | 39 | Periductal | IGHV1-69*01 F | 203/220 nt | 7.73 | IGHJ3*02 F | IGHD3-3*01 F | CAREIFQPGAFDIW |
| pSS2 | 40 | Periductal | IGHV1-69*01 F | 207/220 nt | 5.91 | IGHJ3*02 F | IGHD3-9*01 F | CAREIFQPGAFDIW |
| pSS2 | 41 | Periductal | IGHV1-69*01 F | 210/220 nt | 4.55 | IGHJ3*02 F | IGHD3-3*01 F | CAREIFQPGAFDIW |
| pSS2 | 42 | Periductal | IGHV1-69*01 F | 207/220 nt | 5.91 | IGHJ3*02 F | IGHD3-9*01 F | CAREIFQPGAFDIW |
| pSS2 | 43 | Periductal | IGHV1-69*01 F | 210/220 nt | 4.55 | IGHJ3*02 F | IGHD3-3*01 F | CAREIFQPGAFDIW |
| pSS2 | 44 | Periductal | IGHV1-69*01 F | 210/220 nt | 4.55 | IGHJ3*02 F | IGHD3-9*01 F | CAREIFQPGAFDIW |
| pSS2 | 45 | Periductal | IGHV1-69*01 F | 203/220 nt | 7.73 | IGHJ3*02 F | IGHD3-3*01 F | CAREIFQPGAFDIW |
| pSS2 | 46 | Periductal | IGHV1-69*01 F | 207/220 nt | 5.91 | IGHJ3*02 F | IGHD3-9*01 F | CAREIFQPGAFDIW |
| pSS2 | 47 | Periductal | IGHV1-69*01 F | 202/220 nt | 8.18 | IGHJ3*02 F | IGHD3-3*01 F | CAREIFQPGAFDIW |
| pSS2 | 48 | Periductal | IGHV1-69*01 F | 209/220 nt | 5.00 | IGHJ3*02 F | IGHD3-3*01 F | CAREIFQPGAFDIW |
| pSS2 | 49 | Periductal | IGHV1-69*01 F | 210/220 nt | 4.55 | IGHJ3*01 F | IGHD3-9*01 F | CAREIFQPGAFDLW |
| pSS2 | 50 | Periductal | IGHV1-69*01 F | 210/220 nt | 4.55 | IGHJ3*02 F | IGHD3-9*01 F | CAREIFQPGAFDTW |
| pSS2 | 51 | Periductal | IGHV1-69*01 F | 209/220 nt | 5.00 | IGHJ3*01 F | IGHD3-9*01 F | CAREIFQPGAFDVW |
| pSS2 | 52 | Periductal | IGHV1-69*01 F | 219/219 nt | 0.00 | IGHJ4*02 F | IGHD6-13*01 F | CARTGRGIAAHYDYW |
| pSS2 | 53 | Periductal | IGHV5-10-1*01 F | 219/222 nt | 1.35 | IGHJ3*02 F | IGHD5-18*01 F | CAAPAYCYGSYAFDIW |
|  |  |  |  |  |  |  |  |  |
| pSS3 | 1 | Ductal | IGHV1-18*01 F | 197/220 nt | 10.45 | IGHJ6*02 F | IGHD3-16*01 F | CARDKGWNVLGGYDSFGMDVW |
| pSS3 | 2 | Ductal | IGHV1-18*01 F | 196/220 nt | 10.91 | IGHJ6*02 F | IGHD3-16*01 F | CARDKGWNVLGGYDSFGMDVW |
| pSS3 | 3 | Ductal | IGHV1-18*01 F | 196/220 nt | 10.91 | IGHJ6*02 F | IGHD3-16*01 F | CARDKGWNVLGGYDSFGMDVW |
| pSS3 | 4 | Ductal | IGHV1-18*01 F | 196/220 nt | 10.91 | IGHJ6*02 F | IGHD3-16*01 F | CARDKGWNVLGGYDSFGMDVW |
| pSS3 | 5 | Ductal | IGHV1-18*01 F | 196/220 nt | 10.91 | IGHJ6*02 F | IGHD3-16*01 F | CARDKGWNVLGGYDSFGMDVW |
| pSS3 | 6 | Ductal | IGHV1-18*01 F | 197/220 nt | 10.45 | IGHJ5*02 F | IGHD6-13*01 F | CARDPYSITAPATSDLW |
| pSS3 | 7 | Ductal | IGHV1-18*01 F | 203/220 nt | 7.73 | IGHJ6*02 F | IGHD2-2*01 F | CARGHTFAYQLLLKGMDVW |
| pSS3 | 8 | Ductal | IGHV1-18*01 F | 202/220 nt | 8.18 | IGHJ6*02 F | IGHD2-2*01 F | CARGHTFAYQLLLKGMDVW |
| pSS3 | 9 | Ductal | IGHV1-69*01 F | 206/220 nt | 6.36 | IGHJ4*02 F | IGHD3-10*01 F | CARDMGSGSGSYYFYW |
| pSS3 | 10 | Ductal | IGHV1-8*02 F | 195/220 nt | 11.36 | IGHJ4*02 F | IGHD4-17*01 F | CVRGRNDFGEW |
| pSS3 | 11 | Ductal | IGHV1-8*02 F | 195/220 nt | 11.36 | IGHJ4*02 F | IGHD4-17*01 F | CVRGRNDFGEW |
| pSS3 | 12 | Ductal | IGHV1-8*02 F | 195/220 nt | 11.36 | IGHJ4*02 F | IGHD4-17*01 F | CVRGRNDFGEW |
| pSS3 | 13 | Ductal | IGHV1-8*02 F | 195/219 nt | 10.96 | IGHJ4*02 F | IGHD4-17*01 F | CVRGRNDFGEW |
| pSS3 | 14 | Ductal | IGHV1-8*02 F | 195/220 nt | 11.36 | IGHJ4*02 F | IGHD4-17*01 F | CVRGRNDFGEW |
| pSS3 | 15 | Ductal | IGHV3-48*01 F | 207/223 nt | 7.17 | IGHJ5*02 F | IGHD5-12*01 F | CARDTLSYSGYDSNEIDPW |
| pSS3 | 16 | Ductal | IGHV4-59*01 F | 217/220 nt | 1.36 | IGHJ6*02 F | IGHD3-10*01 F | CATAFWNYDYGMDVW |
| pSS3 | 17 | Ductal | IGHV4-59*08 F | 184/220 nt | 16.36 | IGHJ5*02 F | IGHD1-1*01 F | CARRVTGTTRGHDYFDLW |
| pSS3 | 18 | Ductal | IGHV4-59*08 F | 189/220 nt | 14.09 | IGHJ4*02 F | IGHD1-20*01 F | CARRITGTTRGHDYFDLW |
| pSS3 | 19 | Ductal | IGHV4-59*08 F | 202/220 nt | 8.18 | IGHJ5*02 F | IGHD1-1*01 F | CARRVTGTTRGHDWFDPW |
| pSS3 | 20 | Ductal | IGHV4-59*08 F | 190/220 nt | 13.64 | IGHJ4*02 F | IGHD1-1*01 F | CARRVTGTTRAHDYFDLW |
| pSS3 | 21 | Ductal | IGHV4-59*08 F | 186/220 nt | 15.45 | IGHJ4*02 F | IGHD1-20*01 F | CARRITGTTRGHDYFDLW |
| pSS3 | 22 | Periductal | IGHV1-18*01 F | 194/220 nt | 11.82 | IGHJ4*02 F | IGHD2-8*01 F | CAADKGFHYFDYW |
| pSS3 | 23 | Periductal | IGHV1-18*01 F | 190/220 nt | 13.64 | IGHJ4*02 F | IGHD5-24*01 ORF | CARDEGLNYLDLW |
| pSS3 | 24 | Periductal | IGHV1-18*01 F | 192/220 nt | 12.73 | IGHJ4*02 F | IGHD4-17*01 F | CVRGRNDFGEW |
| pSS3 | 25 | Periductal | IGHV1-18*01 F | 196/220 nt | 10.91 | IGHJ4*02 F | IGHD4-17*01 F | CVRGRNDFGEW |
| pSS3 | 26 | Periductal | IGHV1-8*01 F | 209/220 nt | 5.00 | IGHJ4*02 F | IGHD6-6*01 F | CARDGSSKWGLSYW |
| pSS3 | 27 | Periductal | IGHV1-8*01 F | 208/220 nt | 5.45 | IGHJ4*02 F | IGHD6-6*01 F | CARDGSSKWGLSYW |
| pSS3 | 28 | Periductal | IGHV1-8*02 F | 208/220 nt | 5.45 | IGHJ4*02 F | IGHD6-6*01 F | CARDGSSKWGLSYW |
| pSS3 | 29 | Periductal | IGHV1-2*02 F | 195/220 nt | 11.36 | IGHJ4*02 F | IGHD6-6*01 F | CVSFPGVPKHPYW |
| pSS3 | 30 | Periductal | IGHV1-2*02 F | 196/220 nt | 10.91 | IGHJ4*02 F | IGHD6-6*01 F | CVSFPGVPKHPYW |
| pSS3 | 31 | Periductal | IGHV1-2*02 F | 187/217 nt | 13.82 | IGHJ4*02 F | IGHD2-8*01 F | CARDGPPYCAQGVCYDQW |
| pSS3 | 32 | Periductal | IGHV1-69*04 F | 194/220 nt | 11.82 | IGHJ3*02 F | IGHD6-13*01 F | CARDEIAAVGTWNDAFDIW |
| pSS3 | 33 | Periductal | IGHV3-49*03 F | 211/229 nt | 7.86 | IGHJ3*01 F | IGHD1-7*01 F | CAKVFGSNWNYAACDHW |
| pSS3 | 34 | Periductal | IGHV3-74*01 F | 196/223 nt | 12.11 | IGHJ4*02 F | IGHD6-13*01 F | CAGEKDAPPYSSRMEYW |
| pSS3 | 35 | Periductal | IGHV3-9*01 F | 219/247 nt | 11.34 | IGHJ4*02 F | IGHD2-8*02 F | CLKETSPGGLDHW |
| pSS3 | 36 | Periductal | IGHV4-59*01 F | 210/220 nt | 4.55 | IGHJ2*01 F | IGHD2-15*01 F | CARDQYCPGGSCFDWYFDVW |
| pSS3 | 37 | Periductal | IGHV4-59*04 F | 184/220 nt | 16.36 | IGHJ5*02 F | IGHD1-1*01 F | CARRVTGTTRGHDYFDLW |
| pSS3 | 38 | Periductal | IGHV4-59*08 F | 200/220 nt | 9.09 | IGHJ6*02 F | IGHD3-9*01 F | CARRPPPLTGPYIHFYGMDVW |
| pSS3 | 39 | Periductal | IGHV5-51*01 F | 215/222 nt | 3.15 | IGHJ6*02 F | IGHD5-18*01 F | CARLDTSMTYYYYSHMDVW |
|  |  |  |  |  |  |  |  |  |
| pSS4 | 1 | Ductal | IGHV1-69*01 F | 202/219 nt | 7.76 | IGHJ4*02 F | IGHD2-21*01 F | CARGTGEGGGYYYDYW |
| pSS4 | 2 | Ductal | IGHV3-21*01 F | 196/223 nt | 12.11 | IGHJ4*02 F | IGHD1-14*01 ORF | CSNGYREQLSGSGPGSFFDYW |
| pSS4 | 3 | Ductal | IGHV3-21*01 F | 197/223 nt | 11.66 | IGHJ4*02 F | IGHD1-14*01 ORF | CSNGYREQLSGSGPGSFFDYW |
| pSS4 | 4 | Ductal | IGHV3-23*01 F | 190/213 nt | 10.80 | IGHJ5*02 F | IGHD2-8*01 F | CVRDRLYCSNGVCFEHLFDPW |
| pSS4 | 5 | Ductal | IGHV3-23*01 F | 201/223 nt | 9.87 | IGHJ5*02 F | IGHD2-8*01 F | CVRDRLYCSNGVCFEHLFDPW |
| pSS4 | 6 | Ductal | IGHV3-23*01 F | 200/223 nt | 10.31 | IGHJ5*02 F | IGHD2-8*01 F | CVRDRLYCSNGVCFEHLFDPW |
| pSS4 | 7 | Ductal | IGHV3-23*01 F | 200/223 nt | 10.31 | IGHJ5*02 F | IGHD2-8*01 F | CVRDRLYCSNGVCFEHLFDPW |
| pSS4 | 8 | Ductal | IGHV3-23*01 F | 200/223 nt | 10.31 | IGHJ5*02 F | IGHD2-8*01 F | CVRDRLYCSNGVCFEHLFDPW |
| pSS4 | 9 | Ductal | IGHV3-23*01 F | 201/223 nt | 9.87 | IGHJ5*02 F | IGHD2-8*01 F | CVRDRLYCSNGVCFEHLFDPW |
| pSS4 | 10 | Ductal | IGHV3-23*05 F | 194/223 nt | 13.00 | IGHJ4*02 F | IGHD3-9*01 F | CAKGSVNIWCLDSW |
| pSS4 | 11 | Ductal | IGHV3-23*05 F | 195/223 nt | 12.56 | IGHJ4*02 F | IGHD3-9*01 F | CAKGSVNIWCLDSW |
| pSS4 | 12 | Periductal | IGHV1-18*01 F | 202/219 nt | 7.76 | IGHJ5*02 F | IGHD4-17*01 F | CARDRDYGDYVDLGWFDPW |
| pSS4 | 13 | Periductal | IGHV1-18*01 F | 219/219 nt | 0.00 | IGHJ5*02 F | IGHD3-10*01 F | CARERPLVRGPGGWFDPW |
| pSS4 | 14 | Periductal | IGHV1-18*01 F | 218/219 nt | 0.46 | IGHJ5*02 F | IGHD3-10*01 F | CARERPLVRGPGGWFDPW |
| pSS4 | 15 | Periductal | IGHV1-18*01 F | 188/219 nt | 14.16 | IGHJ4*02 F | IGHD3-22*01 F | CARGGGVDYYDTNGYRFDYW |
| pSS4 | 16 | Periductal | IGHV1-18*04 F | 186/219 nt | 15.07 | IGHJ1*01 F | IGHD2-2*01 F | CARVRTSVAEEFLEYW |
| pSS4 | 17 | Periductal | IGHV3-15*01 F | 206/229 nt | 10.04 | IGHJ4*02 F | IGHD5-24*01 ORF | CAHDDYHFSGFW |
| pSS4 | 18 | Periductal | IGHV3-15*01 F | 207/229 nt | 9.61 | IGHJ4*02 F | IGHD5-24*01 ORF | CAHDDYHFSGFW |
| pSS4 | 19 | Periductal | IGHV3-33*01 F | 202/223 nt | 9.42 | IGHJ4*02 F | IGHD6-19*01 F | CARVGSVADSFDYW |
| pSS4 | 20 | Periductal | IGHV3-7*01 F | 207/223 nt | 7.17 | IGHJ6*02 F | IGHD3-10*01 F |  |
| pSS4 | 21 | Periductal | IGHV4-39*01 F | 219/226 nt | 3.10 | IGHJ3*01 F | IGHD3-9*01 F | CARHGLVTLSPRDGFDFW |
| pSS4 | 22 | Periductal | IGHV4-39*01 F | 220/227 nt | 3.08 | IGHJ3*01 F. | IGHD3-9*01 F | CARHGLVTLSPRDGFDFW |
| pSS4 | 23 | Periductal | IGHV4-39*01 F | 215/227 nt | 5.29 | IGHJ4*02 F | IGHD6-25*01 F | CATYVEHWLQEPADW |
| pSS4 | 24 | Periductal | IGHV4-39*02 F | 201/226 nt | 11.06 | IGHJ4*02 F | IGHD2-2*02 F | CGRLNSDCSIIPCYKGYFDSW |
| pSS4 | 25 | Periductal | IGHV4-39*02 F | 202/226 nt | 10.62 | IGHJ4*02 F | IGHD2-2*02 F | CGRLNSDCSIIPCYKGYFDSW |
| pSS4 | 26 | Periductal | IGHV4-59*01 F | 193/221 nt | 12.67 | IGHJ6*02 F | IGHD6-19*01 F | CARGGSGAVAGTRPFLSYGMDVW |
| pSS4 | 27 | Periductal | IGHV4-59*01 F | 188/221 nt | 14.93 | IGHJ4*02 F | IGHD4-11*01 ORF | CATADYPNIRWGSDSW |
|  |  |  |  |  |  |  |  |  |
| pSS5 | 1 | Ductal | IGHV1-2*02 F | 203/219 nt | 7.31 | IGHJ3*01 F | IGHD3-3*02 F | CAKTHGLIRIFGVMVNRDGFDFW |
| pSS5 | 2 | Ductal | IGHV1-2*02 F | 202/219 nt | 7.76 | IGHJ3*01 F | IGHD3-3*02 F | CAKTHGLIRIFGVMVNRDGFDFW |
| pSS5 | 3 | Ductal | IGHV1-2*02 F | 186/219 nt | 15.07 | IGHJ4*02 F | IGHD4-23*01 ORF | CATGGDDGGESGVLFYW |
| pSS5 | 4 | Ductal | IGHV1-2*02 F | 185/219 nt | 15.53 | IGHJ4*02 F | IGHD4-23*01 ORF | CATGGDDGGESGVLFYW |
| pSS5 | 5 | Ductal | IGHV1-69*01 F | 189/218 nt | 13.30 | IGHJ4*02 F | IGHD2-21*01 F | CARGTGEGGGYYYDYW |
| pSS5 | 6 | Ductal | IGHV1-69*01 F | 200/219 nt | 8.68 | IGHJ4*02 F | IGHD2-21*01 F | CARGTGEGGGYYYDYW |
| pSS5 | 7 | Ductal | IGHV1-69*01 F | 202/219 nt | 7.76 | IGHJ4*02 F | IGHD2-21*01 F | CARGTGEGGGYYYDYW |
| pSS5 | 8 | Ductal | IGHV1-69*01 F | 202/219 nt | 7.76 | IGHJ4*02 F | IGHD2-21*01 F | CARGTGEGGGYYYDYW |
| pSS5 | 9 | Ductal | IGHV1-69*01 F | 201/219 nt | 8.22 | IGHJ4*02 F | IGHD2-21*01 F | CARGTGEGGGYYYDYW |
| pSS5 | 10 | Ductal | IGHV1-69*01 F | 201/219 nt | 8.22 | IGHJ4*02 F | IGHD2-21*01 F | CARGTGEGGGYYYDYW |
| pSS5 | 11 | Ductal | IGHV1-69*01 F | 201/219 nt | 8.22 | IGHJ4*02 F | IGHD2-21*01 F | CARGTGEGGGYYYDYW |
| pSS5 | 12 | Ductal | IGHV1-69*01 F | 202/219 nt | 7.76 | IGHJ4*02 F | IGHD3-16*01 F | CARGTAGSFGYYYDYW |
| pSS5 | 13 | Ductal | IGHV1-69*01 F | 215/219 nt | 1.83 | IGHJ4*02 F | IGHD4-23*01 ORF | CAREGKGNDYGGNPFDYW |
| pSS5 | 14 | Ductal | IGHV3-74*01 F | 207/223 nt | 7.17 | IGHJ6*02 F | IGHD6-19*01 F | CARDSLLSQWLELRYGMDVW |
| pSS5 | 15 | Ductal | IGHV4-59*03 F | 196/220 nt | 10.91 | IGHJ6*02 F | IGHD3-9*01 F |  |
| pSS5 | 16 | Periductal | IGHV1-2*02 F | 191/219 nt | 12.79 | IGHJ5*02 F | No results |  |
| pSS5 | 17 | Periductal | IGHV1-2*02 F | 197/219 nt | 10.05 | IGHJ4*02 F | IGHD3-3*02 F |  |
| pSS5 | 18 | Periductal | IGHV1-69*01 F | 207/219 nt | 5.48 | IGHJ4*02 F | IGHD6-13*01 F |  |
| pSS5 | 19 | Periductal | IGHV1-69*01 F | 206/219 nt | 5.94 | IGHJ4*02 F | IGHD6-13*01 F |  |
| pSS5 | 20 | Periductal | IGHV1-69*01 F | 212/219 nt | 3.20 | IGHJ4*02 F | IGHD4-23*01 ORF |  |
| pSS5 | 21 | Periductal | IGHV1-69*01 F | 215/219 nt | 1.83 | IGHJ4*02 F | IGHD4-23*01 ORF |  |
| pSS5 | 22 | Periductal | IGHV1-69*01 F | 214/219 nt | 2.28 | IGHJ4*02 F | IGHD4-23*01 ORF |  |
| pSS5 | 23 | Periductal | IGHV1-69*01 F | 211/219 nt | 3.65 | IGHJ4*02 F | IGHD4-23*01 ORF |  |
| pSS5 | 24 | Periductal | IGHV1-69*01 F | 206/219 nt | 5.94 | IGHJ4*02 F | IGHD4-23*01 ORF |  |
| pSS5 | 25 | Periductal | IGHV1-69*01 F | 214/219 nt | 2.28 | IGHJ4*02 F | IGHD4-23*01 ORF |  |
| pSS5 | 26 | Periductal | IGHV1-69*01 F | 204/219 nt | 6.85 | IGHJ4*02 F | IGHD3-10*01 F |  |
| pSS5 | 27 | Periductal | IGHV1-69*01 F | 203/219 nt | 7.31 | IGHJ4*02 F | IGHD3-10*01 F |  |
| pSS5 | 28 | Periductal | IGHV1-69*01 F | 194/219 nt | 11.42 | IGHJ6*02 F | IGHD2-2*01 F |  |
| pSS5 | 29 | Periductal | IGHV1-69*01 F | 193/219 nt | 11.87 | IGHJ6*02 F | IGHD2-2*01 F |  |
| pSS5 | 30 | Periductal | IGHV1-69*01 F | 193/219 nt | 11.87 | IGHJ6*02 F | IGHD2-2*01 F |  |
| pSS5 | 31 | Periductal | IGHV1-8*01 F | 197/219 nt | 10.05 | IGHJ5*02 F | IGHD6-13*01 F |  |
| pSS5 | 32 | Periductal | IGHV1-8*01 F | 213/219 nt | 2.74 | IGHJ6*02 F | IGHD3-10*01 F |  |
| pSS5 | 33 | Periductal | IGHV1-8*01 F | 212/219 nt | 3.20 | IGHJ6*02 F | IGHD3-10*01 F |  |
| pSS5 | 34 | Periductal | IGHV3-23*01 F | 194/223 nt | 13.00 | IGHJ4*02 F | IGHD3-22*01 F |  |
| pSS5 | 35 | Periductal | IGHV3-23*01 F | 212/223 nt | 4.93 | IGHJ4*02 F | IGHD2-15*01 F |  |
| pSS5 | 36 | Periductal | IGHV3-73*01 F | 213/229 nt | 6.99 | IGHJ3*01 F | IGHD2-15*01 F |  |
| pSS5 | 37 | Periductal | IGHV4-61*03 F | 193/226 nt | 14.60 | IGHJ5*01 F | IGHD1-26*01 F |  |
| pSS5 | 38 | Periductal | IGHV5-10-1*01 F | 200/222 nt | 9.91 | IGHJ4*02 F | IGHD5-24*01 ORF |  |
| pSS5 | 39 | Periductal | IGHV5-51*01 F | 218/222 nt | 1.80 | IGHJ6*02 F | IGHD6-13*01 F |  |

Supplementary Table 2. Summary of IMGT/V-Quest analysis of all IGHV sequences microdissected from ductal areas and periductal areas.
