# Supplementary figures and images for: Repertoire Analysis of B-Cells Located in Striated Ducts of Salivary Glands of Patients With Sjögren's Syndrome
Source: Front Immunol. 2020 Jul 14;11:1486. doi: 10.3389/fimmu.2020.01486 (PMC7372116; doi:10.3389/fimmu.2020.01486)

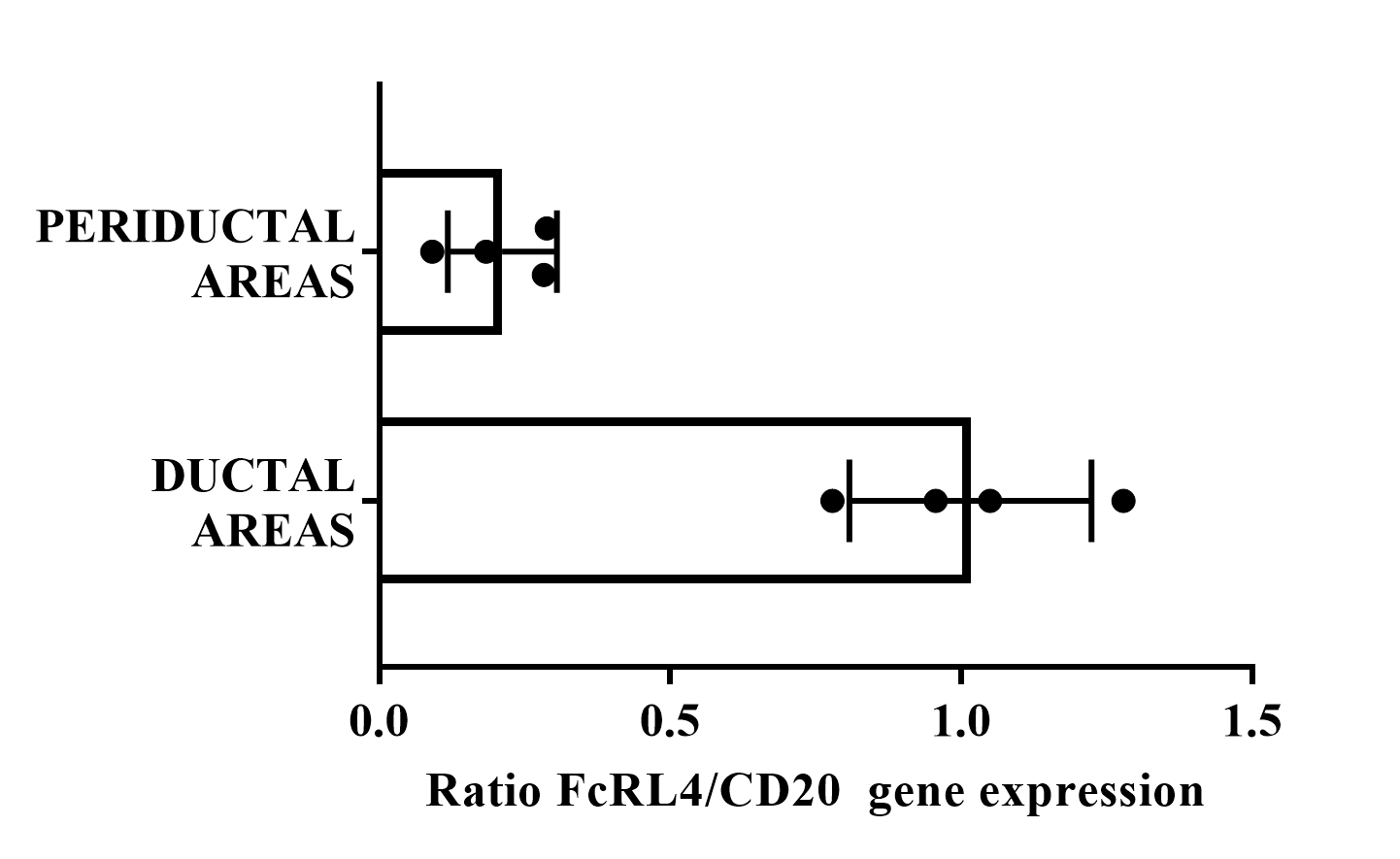

Supplement: Supplementary file 4 [file Image_1.tif]

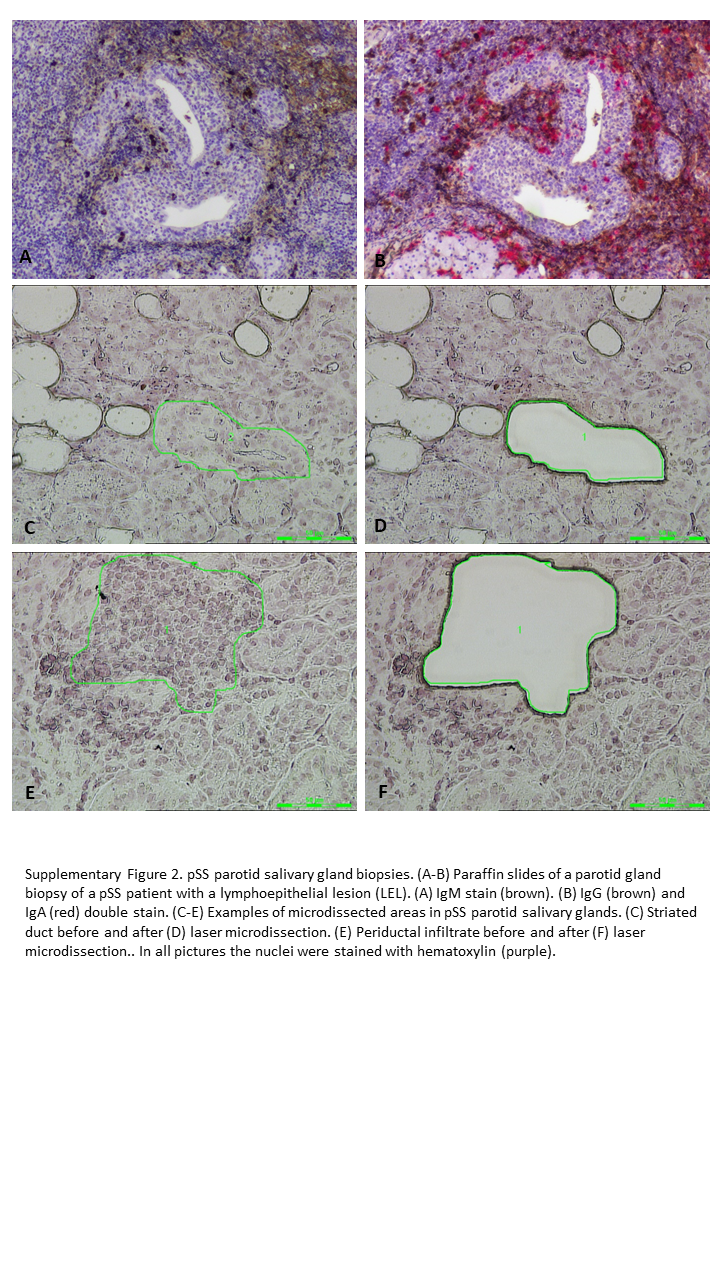

Supplement: Supplementary file 5 [file Image_2.tif]
